# Supplementary material for: IFN signaling and neutrophil degranulation transcriptional signatures are induced during SARS-CoV-2 infection
Source: Commun Biol. 2021 Mar 5;4:290. doi: 10.1038/s42003-021-01829-4 (PMC7935909; doi:10.1038/s42003-021-01829-4)
Supplement: Supplementary file 1 — Supplementary Information [file 42003_2021_1829_MOESM1_ESM.pdf]

Supplementary Table 1: Significant Reactome and KEGG pathway enrichment among the 1,026 genes significantly upregulated by COVID-19.

Pathway ID	Pathway description	Total pathway size	# Significant genes	FDR-corrected P value
**Reactome**				
R-HSA-6798695	Neutrophil degranulation	479	65	1.1E-07
R-HSA-168256	Immune System	1997	162	1.0E-03
R-HSA-168249	Innate Immune System	1053	95	3.1E-03
R-HSA-162906	HIV Infection	232	31	4.3E-03
R-HSA-72766	Translation	291	36	4.3E-03
R-HSA-162587	HIV Life Cycle	151	22	0.016
R-HSA-5368287	Mitochondrial translation	93	16	0.019
R-HSA-8953854	Metabolism of RNA	673	63	0.019
R-HSA-72306	tRNA processing	106	17	0.021
R-HSA-5389840	Mitochondrial translation elongation	87	15	0.021
R-HSA-162599	Late Phase of HIV Life Cycle	138	20	0.021
R-HSA-9018679	Biosynthesis of EPA-derived SPMs	6	4	0.024
R-HSA-913531	Interferon Signaling	197	25	0.024
R-HSA-8978868	Fatty acid metabolism	177	23	0.027
R-HSA-6781823	Formation of TC-NER Pre-Incision Complex	54	11	0.027
R-HSA-5696399	Global Genome Nucleotide Excision Repair (GG-NER)	84	14	0.034
R-HSA-159231	Transport of Mature mRNA Derived from an Intron less Transcript	40	9	0.040
R-HSA-5419276	Mitochondrial translation termination	87	14	0.044
R-HSA-159234	Transport of Mature mRNAs Derived from Intron less Transcripts	41	9	0.044
R-HSA-1236975	Antigen processing-Cross presentation	99	15	0.048
R-HSA-6781827	Transcription-Coupled Nucleotide Excision Repair (TC-NER)	79	13	0.048
**KEGG**				
KEGG:01100	Metabolic pathways	1391	131	1.9E-04
KEGG:00020	Citrate cycle (TCA cycle)	32	10	2.4E-03
KEGG:03060	Protein export	24	8	0.010
KEGG:01212	Fatty acid metabolism	55	12	0.018
KEGG:00280	Valine, leucine and isoleucine degradation	48	11	0.021

Supplementary Table 2: Significant Reactome and KEGG pathway enrichment among the 1,109 genes significantly downregulated by COVID-19.

Pathway ID	Pathway description	Total pathway size	# Significant genes	FDR-corrected P value
**Reactome**				
R-HSA-5653656	Vesicle-mediated transport	667	74	2.5E-05
R-HSA-199991	Membrane Trafficking	628	66	6.1E-04
R-HSA-1442490	Collagen degradation	64	14	6.4E-03
R-HSA-73887	Death Receptor Signaling	141	22	6.4E-03
R-HSA-194315	Signaling by Rho GTPases	444	47	7.8E-03
R-HSA-8948216	Collagen chain trimerization	44	11	7.8E-03
R-HSA-2022090	Assembly of collagen fibrils and other multimeric structures	61	13	7.9E-03
R-HSA-1474290	Collagen formation	90	16	9.5E-03
R-HSA-170834	Signaling by TGF-beta Receptor Complex	73	14	0.011
R-HSA-1650814	Collagen biosynthesis and modifying enzymes	67	13	0.013
R-HSA-3247509	Chromatin modifying enzymes	275	32	0.013
R-HSA-4839726	Chromatin organization	275	32	0.013
R-HSA-194840	Rho GTPase cycle	138	20	0.014
R-HSA-2214320	Anchoring fibril formation	15	6	0.014
R-HSA-9006934	Signaling by Receptor Tyrosine Kinases	455	45	0.022
R-HSA-9006936	Signaling by TGF-beta family members	102	16	0.022
R-HSA-446353	Cell-extracellular matrix interactions	18	6	0.033
R-HSA-2243919	Crosslinking of collagen fibrils	18	6	0.033
R-HSA-1474228	Degradation of the extracellular matrix	140	19	0.033
R-HSA-193704	p75 NTR receptor-mediated signaling	97	15	0.033
R-HSA-193648	NRAGE signals death through JNK	59	11	0.037
R-HSA-416482	G alpha (12/13) signaling events	79	13	0.038
R-HSA-3000480	Scavenging by Class A Receptors	19	6	0.038
R-HSA-5140745	WNT5A-dependent internalization of FZD2, FZD5 and ROR2	13	5	0.040
R-HSA-9007101	Rab regulation of trafficking	124	17	0.047
**KEGG**				
KEGG:04144	Endocytosis	231	37	3.0E-06
KEGG:05165	Human papillomavirus infection	314	42	6.3E-05
KEGG:04510	Focal adhesion	194	29	4.3E-04
KEGG:04530	Tight junction	150	24	9.1E-04
KEGG:05135	Yersinia infection	116	20	1.7E-03
KEGG:05132	Salmonella infection	205	26	0.023
KEGG:04390	Hippo signaling pathway	146	20	0.045

Supplementary Table 3: Significant Reactome and KEGG pathway enrichment among the 86 genes significantly upregulated by COVID-19 only in Juvenile macaques.

Pathway ID	Pathway description	Total pathway size	# Significant genes	FDR-corrected P value
**Reactome**				
R-HSA-909733	Interferon alpha/beta signaling	69	5	0.033
**KEGG**				
KEGG:04330	Notch signaling pathway	53	4	9.9E-03
KEGG:05160	Hepatitis C	140	5	0.047

Supplementary Table 4: Significant Reactome and KEGG pathway enrichment among the 160 genes significantly downregulated by COVID-19 only in Old macaques.

Pathway ID	Pathway description	Total pathway size	# Significant genes	FDR-corrected P value
**Reactome**				
R-HSA-4420097	VEGFA-VEGFR2 Pathway	99	7	0.037
R-HSA-194138	Signaling by VEGF	107	7	0.037
**KEGG**				
KEGG:04611	Platelet activation	122	8	2.5E-03
KEGG:05206	MicroRNAs in cancer	158	8	0.016
