# Supplementary material for: IFN signaling and neutrophil degranulation transcriptional signatures are induced during SARS-CoV-2 infection
Source: Commun Biol. 2021 Mar 5;4:290. doi: 10.1038/s42003-021-01829-4 (PMC7935909; doi:10.1038/s42003-021-01829-4)
Supplement: Supplementary file 2 — Description of Additional Supplementary Files [file 42003_2021_1829_MOESM2_ESM.pdf]

**Description of Additional Supplementary Files**

**File name: Supplementary Data 1**

**Description:** Read processing and mapping statistics, and download accessions for all RNA-seq samples and clinical data from SARS-CoV-2 infected macaques.

**File name: Supplementary Data 2**

**Description:** Fragment counts, relative gene expression levels, gene annotations, and differential expression data for every macaque gene.

**File name: Supplementary Data 3**

**Description:** Complete lists of significantly differentially expressed gene sets of interest (including gene names, relative expression data, fold change and P values). Gene sets include: (**A**) 1,026 genes significantly upregulated with COVID-19 vs Naive, (**B**) 65 "neutrophil degranulation" (R-HSA-6798695) genes significantly upregulated during COVID-19, (**C**) 162 "neutrophil degranulation" (R-HSA-6798695) genes significantly upregulated during COVID-19, (**D**) 1,109 genes significantly downregulated with COVID-19 vs Naive, (**E**) 14 "collagen degradation" (R-HSA-1442490) genes significantly downregulated during COVID-19, (**F**) 14 "Signaling by TGF-beta Receptor Complex" (R-HSA-170834) genes significantly downregulated during COVID-19, (**G**) 86 genes significantly upregulated with COVID-19 vs Naive only in Juvenile macaques, (**H**) 96 genes significantly downregulated with COVID-19 vs Naive only in Juvenile macaques, (**I**) 97 genes significantly upregulated with COVID-19 vs Naive only in Old macaques, (**J**) 160 genes significantly downregulated with COVID-19 vs Naive only in Old macaques, (**K**) 97

genes significantly upregulated by both COVID-19 and TB and (**L**) 76 genes significantly upregulated by COVID-19 but downregulated by TB.

**File name: Supplementary Data 4**

**Description:** Significant functional enrichment for Reactome, KEGG and Gene Ontology pathways, among differentially gene sets of interest. Gene sets include: (**A**) 1,026 genes upregulated in COVID-19 vs Naive, (**B**) 1,109 genes downregulated in COVID-19 vs Naive, (**C**) 86 genes signficantly upregulated by COVID-19 only in Juvenile macaques, (**D**) 160 genes signficantly downregulated by COVID-19 only in Old macaques, (**E**) 97 genes significantly upregulated by both COVID-19 and TB, and (**F**) 76 genes significantly upregulated by COVID-19 but downregulated by TB.

**File name: Supplementary Data 5**

**Description:** (**A**) Clinical characteristics and lab parameters of COVID-19 patients. Clinical and demographic data were retrieved from the medical records of all participants. These data included age, gender, anthropometrics, comorbidities, symptoms, triage vital signs, and initial laboratory test results (**B**) Levels of IFNγ (**C**) Levels of VEGF (**D**) Neutrophil counts and (**E**) NLR values from plasma and blood samples of COVID-19 patients.

**File name: Supplementary Data 6**

**Description:** Clinical data of TB-infected macaques.
